# 

**Published:** 1907-11-30

**Authors:** 


### THE PLAGUE IN SYDNEY.

For seven years Sydney has paid an annual tribute to the bubonic plague, and this year the disease has once more made its appearance, so that it seems as if it had gained a permanent footing in the city in spite of the active propaganda of prophylaxis which the authorities have carried on against it. The total number of cases which have been reported during the last quarter is 30, of which eight have ended fatally. In addition, two doubtful cases were reported, bringing the number up to 32. In most of these cases infection was directly traceable to rats, and an interesting point, referred to by the medical officer in his report, is the fact that in some cases infected rats were found far from the original site of infection, "as the result, probably, of delirious wanderings." There is little information to hand as to the form of plague specially prevalent in Sydney. The majority of fatal cases seem to have been of the septicæmic form, while the greater number of convalescent patients suffered from the bubonic type. This is what one might have expected from the history of former epidemics. The local medical press insists on the necessity of preventing a recrudescence of the disease, rather than on curative measures while the epidemic is active, and ascribes the spread of the plague to the apathy of the public, and to the existence of old rat-infected buildings which continue to remain as menaces to the public health. No attempt seems to have been made to exterminate the rats on a large scale, and indeed it must be admitted that much remains to be done towards perfecting measures for the wholesale destruction of these plague-carriers.

### THE GRADUATES' MEDICAL SCHOOLS.

### Diary for the week December 2 to 7.

**[illegible]HE ROYAL COLLEGE OF PHYSICIANS.**

**[illegible]Patrick Lectures.**

[illegible]ember 3 and 5, Dr. Leonard Guthrie, **Contributions [illegible]rom History and Literature to the Study of [illegible]recocity in Children.**

**[illegible]MEDICAL GRADUATES' COLLEGE AND POLYCLINIC, Chenies Street, W.C.**

**[illegible]ai[illegible]** (except Saturday): Clinics, Demonstrations and Practical classes.

**[illegible]ectures at 5.15 p.m.**

December 2, Mr. P. W. [illegible]G. Sargent, **The Surgical Treatment of Infantile [illegible]aralysis.**

December 3, Dr. M. Dob[illegible]ie, **Swedish Medical Gymnastics and their Ap[illegible]lication in the Treatment of Muscle and Joint Affections, Fractures and Deformities.**

December 4, Dr. Burney [illegible]eo, **Diet in Old Age.**

December 5, Mr. James [illegible]erry, **The Treatment of Hip Disease in its Later Stages.**

**LONDON SCHOOL OF CLINICAL MEDICINE, Seamen's Hospital, Greenwich.**

**Medical and Surgical Clin[illegible]ics.**—Demonstrations and special departments daily.

**Lectures.**

December 2, **3.15 p.m.**, Mr. W[illegible]. Turner, **Treatment of Urethral Stricture.**

December 5, **2.15 p.m.**, Dr. Rank[illegible]in, **Progressive Muscular Atrophy.**

**THE POST-GRADUATE COLLEGE, West London Hospital, Hammersmith, W.**

**Daily at 2:** Medical and Surgical Clinics and $x$-Rays; **at 2.30 p.m.:** Operations.

**Special Departments at 10** (Tuesday, Wednesday, Friday, Saturday), and daily at **2 p.m.**

**Lectures.**

December 2, **12 noon**, Dr. Low, **Pathological Demonstration.**

**At 5 p.m.**

December 2, Mr. Edwards, **Surgical Cases.**
December 3, Dr. Low, **Beri-Beri.**
December 4, Dr. Beddard, **Practical Medicine.**
December 5, Mr. Keetley, **Plastic Surgery.**
December 6, Dr. Seymour Taylor, **Treatment of Common Maladies.**

**WEST END HOSPITAL FOR DISEASES OF THE NERVOUS SYSTEM, Welbeck Street, W.**

**Welbeck Street, 5 p.m.**

December 5, Dr. E. Macnamara, **Epilepsy.**

**NATIONAL HOSPITAL FOR THE PARALYSED AND EPILEPTIC, Queen Square, Bloomsbury, W.C.**

**Lectures at 3.30 p.m.**

December 3, Dr. Aldren Turner, **Muscular Atrophy.**
December 6, Mr. Armour, **Spinal Caries.**

**THE ROYAL DENTAL HOSPITAL.**

**At 5.30 p.m.**

December 2, Mr. J. G. Turner, **The Ætiology of Palatal Deformities.**

### BUSINESS NOTICES.

Letters relating to the Publishing, Sale and Advertisement Departments must be addressed to the Manager (*not to the Editor*):—

THE MANAGER,
The Hospital Building,
28 and 29 Southampton Street,
Strand, London, W.C.

**Annual Subscriptions.**

The rate of annual subscriptions to THE HOSPITAL, either direct from the Office or through agents, is:—

For the United Kingdom ... ... 15s. a year
To the Colonies and Abroad ... 17s. a year
inclusive of postage in each case.

**Subscriptions.**

Subscriptions are payable in advance. Cheques and Postal Orders should be made payable to:—

THE SCIENTIFIC PRESS, LTD., and should be crossed: London and County Banking Co., Ltd.

**Cases for Binding.**

Cases for binding half-yearly volumes of THE HOSPITAL may be obtained from the Manager, price 2s. post free. The Index to Vol. xlii. is now ready, price 3½d., post free. The monthly part for November is also ready, price 1s., by post 1s. 3d.

**Books for Review.**

Publishers are particularly requested to send advance proofs of any new books of importance, whenever possible, as the Editor has made arrangements to publish immediate reviews, and on a new plan.

THE HOSPITAL *November 30, 1907.*

*Name* ..........................................................................................

*Address* ......................................................................................

......................................................................................................

This Coupon must accompany manuscript or contributions intended for THE HOSPITAL.

THE SCIENTIFIC PRESS, LTD.,

28/29 Southampton Street, Strand, London, W.C.

**NOTES ON GENERAL PRACTICE.**

**3/6 *net.*** 3/9 post free.

By S. M. HEBBLETHWAITE, M.D.Lond.

**3/6 *net.*** 3/9 post free

"Dr. Hebblethwaite has set himself to guard the steps of the unwary by a practical description of some of the more frequent pitfalls met with in private practice. His advice is in all cases . . . characterised by method and a sound common sense; while his notes on perforative peritonitis, rheumatism in children, caries of the cervical spine . . . may be specially singled out as being worthy of remembrance."—*The Hospital.*

"Any newly fledged graduate . . . will find it a bridge from hospital practice . . . to the private practice in which he is so often doomed to learn that much of his hardly acquired knowledge is but vanity."—*Edinburgh Medical Journal.*

**A NEW CLINICAL CHART for recording TEMPERATURE, PULSE and RESPIRATION.**

Designed by S. M. HEBBLETHWAITE, M.D.

Size 11½ by 9 in. Price 1/- per dozen; 1/1 post free.

Published by THE SCIENTIFIC PRESS, LTD., 28 and 29 Southampton Street, Strand, London, W.C.

